# Sepsis-related deaths in the at-risk population on the wards: attributable fraction of mortality in a large point-prevalence study

**DOI:** 10.1186/s13104-018-3819-2

**Published:** 2018-10-11

**Authors:** Maja Kopczynska, Ben Sharif, Sian Cleaver, Naomi Spencer, Amit Kurani, Camilla Lee, Jessica Davis, Carys Durie, Jude Joseph-Gubral, Angelica Sharma, Lucy Allen, Billie Atkins, Alex Gordon, Llewelyn Jones, Amy Noble, Matthew Bradley, Henry Atkinson, Joy Inns, Harriet Penney, Carys Gilbert, Rebecca Walford, Louise Pike, Ross Edwards, Robyn Howcroft, Hazel Preston, Jennifer Gee, Nicholas Doyle, Charlotte Maden, Claire Smith, Nik Syakirah Nik Azis, Navrhinaa Vadivale, Tamas Szakmany, Welsh Digital Data Collection Platform Collaborators, Richard Pugh, Ceri Battle, Ronan Lyons, Paul Morgan, Maria Hobrok, Igor Otahal, Peter Havalda, Vincent Hamlyn, Luis Macchiavello, Rhidian Jones, Orsolya Minik, Paul Morgan, Gemma Ellis, Ceri Brown, Chris Littler, Andy Campbell, David George, Chris Subbe, Judith E. Hall, Szilvia Szoke, Richard Self, Una Gunter, Robert M. Lundin, Meshari Alsaeed, Hannah Williams, Arfa Ayob, Nor Farzana, Sweta Parida, David Lawson, Michal Mazur, Lezia D’Souza, Bethan Ponting, Terrance Lau, Ruairidh Kerrigan, Lucy Morgan, Roshan Vindla, Claudia Zeicu, Becky James, Amirah Amin Ariff, Wan Binti Wan Azzlan, Charlotte Collins, Elizabeth Wickens, Alisa Norbee, Aliya Zulkefli, Thomas Haddock, Megan Thomas, Matthew Lee, Akshita Dandawate, Holleh Shayan-Arani, Ellie Taylor, Oliver Kyriakides, Rachel Price, Ffion Haf Mackey, Emily Haines, Samuel Chun, Chantal Roberts, Alessia Waller, Laura Heekin, Kathy Wang, Rhianna Church, Shrina Patel, Marianne Broderick, Hannah Whillis, Daniel Craig Hathaway, Emel Yildirim, Caitlin Atkins, Adam George Mounce, Anoopama Ramjeeawon, Ndaba Mtunzi, Duncan Soppitt, Jay Hale, Jack Wellington, Robert Buchanan Ross, Danielle Lis, Sophie Stovold, Sam Vickery, Nia Jones, Alice O’Donnell, Monty Cuthbert, Osa Eghosa, Muhammad Karim, Lowri Williams, Louise Tucker, Thomas Downs, Ailsa MacNaught, Swagath Balachandran, Abbie Shipley, Jennifer Louise Kent, Talea Roberts, Samuel Tilley, Bethany Davies, Emma Withers, Krishna Parmar, Lucie Webber, Thomas Grother, Harry Smith, Rachel Watson, Natalie Hoyle, Rym Chafai El Alaoui, Omar Marei, Emma Kirby, Anna Gilfedder, Lydia Maw, Sarah O’Connor, Abigail Rogers, Zhao Xuan Tan, Clare Chantrill, Amal Robertson, Jonathan Foulkes, Rahana Khanam, Jomcy John, Isobel Sutherland, Sarah Hannah Meehan, Huria Metezai, Hannah Dawson, Eloise Baxendale, Karishma Khan, Pan Myat, Andrew Forrester, Oliver Moore, Hse Juinn Lim, Aimee Owen, Faris Hussain, Nima-banu Allybocus, Maneha Sethi, Umair Asim, Emelia Boggon, Ibrahim Alkurd, Genevieve Lawrence, Jade Brown, Lowri Hughes Thomas, Emily Murphy, Evie Lambert, Jeremy Guilford, Beth Payne, Mariam Almulaifi, Arwel Poacher, Sashiananthan Ganesananthan, Sara Tanatova, Jasmine Kew, Megan Eilis Clark, Ellen Hannay, Olesya Godsafe, Christina Houghton, Francesca Lavric, Rachel Mallinson, Hei Man Priscilla Chan, Eshen Ang, Niamh McSwiney, Yin Yin Lim, Zong Xuan Lee, Svetlana Kulikouskaya, Nur Zulkifli, Sheryl Lim, Lim Xin, Thomas Chandy, Abduahad Taufik, James Cochrane, Sioned Davies, Samuel Willis, John Lynch, Sieh Yen Heng, Alex Cooper, Henrik Graf von der Pahlen, Isabella Talbot, Robin Gwyn Roberts, Jessica Sharma Smith, Aisling Sweeney, Cerian Roberts, Paul McNulty, Elin Walters, Robert Sinnerton, Benjamin Tanner, Berenice Cunningham-Walker, Chloe Spooner, Akanksha Kiran, Nabeegh Nadeem, Vidhi Unadkat, John Ng Cho Hui, Esme Sparey, David Li, Jessica Smith, India Corrin, Harry Waring, Adeel Khan, Emily Baker, Mohammad Yahya Amjad, Miriam Cynan, Imogen Hay, Catherine Russell, Joseph Davies, Rebecca Parsonson, Ajitha Arunthavarajah, Jessica Nicholas, Aaron Harris, Tim Burnett, Josephine Raffan Gowar, Sam DeFriend, Helena Jones, Nur Amirah Binti Maliki, Mark Zimmerman, Jessica Webber, Rebecca Phillips, Lauren McCarthy, Lara Wirt, Emily Hubbard, Emily Evans, Laura Jane Davis, Llywela Wyn Davies, Lee Sanders-Crook, Amrit Dhadda, Genna Logue, Isabel Jones, Adiya Urazbayeva, Nur Haslina Ahmad Hanif, Yau Ke Ying, Alice Coleclough, Eilis Higgins, Tze Gee Ng, Sam Booth, Nilarnti Vignarajah, Tessa Chamberlain, Dongying Zhao, Nayanatara Nadeesha Tantirige, John Watts, Amy Prideaux, Amelia Tee, Annabelle Hook, Adam Mounce, Emily Eccles, Kirtika Ramesh, Laura Bausor, Amy Handley, Rebecca Paddock, Lopa Banerjee

**Affiliations:** 1 Department of Anaesthesia, Intensive Care and Pain Medicine, Division of Population Medicine, Cardiff University, Heath Park Campus, Cardiff, CF14 4XN UK; 2 Anaesthetic Directorate, Aneurin Bevan University Health Board, Royal Gwent Hospital, Cardiff Road, Newport, Gwent NP20 2UB UK

**Keywords:** Sepsis, Critical care, Frailty, Mortality

## Abstract

**Objective:**

Sepsis mortality is reported to be high worldwide, however recently the attributable fraction of mortality due to sepsis (AFsepsis) has been questioned. If improvements in treatment options are to be evaluated, it is important to know what proportion of deaths are potentially preventable or modifiable after a sepsis episode. The aim of the study was to establish the fraction of deaths directly related to the sepsis episode on the general wards and emergency departments.

**Results:**

839 patients were recruited over the two 24-h periods in 2016 and 2017. 521 patients fulfilled SEPSIS-3 criteria. 166 patients (32.4%) with sepsis and 56 patients (17.6%) without sepsis died within 90 days. Out of the 166 sepsis deaths 12 (7.2%) could have been directly related to sepsis, 28 (16.9%) possibly related and 96 (57.8%) were not related to sepsis. Overall AFsepsis was 24.1%. Upon analysis of the 40 deaths likely to be attributable to sepsis, we found that 31 patients (77.5%) had the Clinical Frailty Score ≥ 6, 28 (70%) had existing DNA-CPR order and 17 had limitations of care orders (42.5%).

**Electronic supplementary material:**

The online version of this article (10.1186/s13104-018-3819-2) contains supplementary material, which is available to authorized users.

## Introduction

Sepsis is defined as dysregulated host response to infection, resulting in acute organ dysfunction [1]. In the UK sepsis is estimated to be responsible for the deaths of 44,000 people every year and hospitalizations for this condition have more than doubled over the last 10 years [2]. Sepsis mortality is reported to be high worldwide, however recently the attributable fraction of mortality due to sepsis (AFsepsis) has been questioned [3]. Very recently the Society of Critical Care Medicine and the European Society of Intensive Care Medicine expert panel on sepsis and septic shock identified this as a key research question: what proportion of deaths are potentially preventable or modifiable after a sepsis episode [4]?

Currently, clinicians rely mostly on nonspecific physiological and laboratory abnormalities among patients with suspected or definite infection [5, 6]. The lack of reliable diagnostic tools makes it more challenging to identify the predictors of patient mortality, design randomised controlled trials (RCT) and develop effective treatments for this condition [6].

Whilst mortality of patients who have been diagnosed with sepsis on the wards or on the ICU is high [7–10], there is very little information about whether this high mortality rate could be directly attributed to this diagnosis [4].

The primary aim of the study was to establish the fraction of deaths directly related to the sepsis episode on the general wards and emergency departments (ED).

## Main text

### Methods

Secondary analysis of patient episodes was performed on patient population recruited into two annual 24-h point-prevalence studies on the general wards and ED across all Welsh acute hospitals in 2016 and 2017 [7, 8]. Each participating hospital was required to have a 24/7 consultant-level Emergency Department supervision and the facility to admit and treat any acutely unwell patient in order to be included in the study. On the study days we enrolled consecutive patients presenting to the ED or being cared for in an acute in-patient ward setting with NEWS ≥ 3 and suspected or proven infection. Patients were excluded if they were less than 18 years of age or if they were already in a Critical Care environment [7, 8].

The methodology of digital data collection and description of the data collector recruitment and performance during the study have been described in our previous studies [8, 11]. The data were collected from medical and nursing records, focusing on patient demographic data, baseline co-morbidity and frailty (according to the Dalhousie Clinical Frailty Scale), clinical observations, laboratory and radiology data to determine sequential organ failure assessment (SOFA) and systemic inflammatory response syndrome (SIRS) sepsis criteria and involvement of the treating teams (such as critical care input and completion of sepsis care bundles) [12]. Patients were followed up until 90 days after study enrolment. We did not perform *a priory* sample size calculation, but aimed to recruit all eligible patients.

### AFsepsis analysis

Deaths attributable to sepsis were evaluated based on microbiological, radiological and laboratory evidence. Cause of death of non-survivors was stratified into ‘Sepsis related’; ‘Possibly sepsis related’ and ‘Non-sepsis related’ using the criteria detailed in Table 1. Based on medical or nursing evaluation documented in the medical notes, we comprehensibly reviewed each decedent’s clinical course in the hospital. We included microbiological, radiological and laboratory parameters to elucidate if the clinical suspicion of infection, which was the study entry criteria in line with the recent SEPSIS-3 definition, could be confirmed. Where available, we also reviewed the discharge letters, clinical summaries and death certificates to search for any evidence of the death being related to sepsis.

**Table 1:** Criteria used for determining sepsis related, possibly sepsis related and non-sepsis related cause of death

Sepsis related death: all three statements apply
Within 14 days of sepsis episode
Significant OR confirmed infectious changes on radiology OR microbiology within 3 days of index episode
Treated with intravenous antibiotics at the time of index episode
Possibly sepsis related death: at least three out of the six statements apply
Within 30 days of the sepsis episode while in-patient
Significant infection related laboratory results within 3 days of the index episode (CRP/WCC)
Administration of multiple broad-spectrum antibiotics (intravenous or oral)
Clinical symptoms convincing of infective origin (SIRS 3 or more) within 3 days of the index episode
Unclear/unavailable data on infection on radiology OR microbiology
Cause of death is “Sepsis”, “Pneumonia” or other infectious origin on death certificate
Non-sepsis related death: Statements 1 AND 2 PLUS either 3 OR 4 apply
No infective changes on laboratory AND radiology investigations AND negative microbiology
Newly discovered OR progression of advanced malignancy (T4 and above) on radiology or pathology
Death after 30 days of the index episode if points 1 AND 2 apply AND previous limitations on level of care in place at the time of index episode
Death after hospital discharge following the index episode if points 1 AND 2 apply OR Cause of death is non-infectious on the death certificate

*CRP* C-reactive protein, *WCC* white cell count

### Statistical analysis

Categorical variables are described as proportions and are compared using Chi square test. Continuous variables are described as median and inter-quartile range and compared using Mann–Whitney U test. A two-tailed p-value < 0.05 was considered statistically significant. All statistical tests were calculated using SPSS 23.0 (SPSS Inc., Chicago, IL).

### Results

In our study we screened 12,477 patients over the two 24-h study periods in the 14 Welsh hospitals. 839 patients had NEWS ≥ 3 and documented clinical suspicion of infection and were recruited in the study. Baseline characteristics are summarised in Table 2.

**Table 2:** Baseline characteristics of the patients for all recruited patients and comparing the non-survivors with survivors within 90-days

	All patients (n = 839)	Non-survivors (n = 222)	Survivors (n = 617)	P-value
Age, median (range)	73 (18–103)	79.5 (22–103)	70 (18–100)	*< 0.0001*
Sex, male	411 (49%)	120 (54.05%)	291 (47.16%)	0.078
COPD	230 (27.4%)	50 (23.36%)	180 (30.05%)	0.062
Diabetes	173 (20.6%)	64 (29.91%)	109 (18.20%)	*0.0003*
Drug abuse	13 (1.5%)	0 (0%)	13 (2.17%)	*0.03*
Heart failure	94 (11.2%)	43 (20.09%)	51 (8.51%)	*< * *0.0001*
Hypertension	272 (32.4%)	76 (35.51%)	196 (32.72%)	0.457
Ischaemic heart disease	145 (17.3%)	46 (21.5%)	99 (16.53%)	0.103
Liver disease	24 (2.9%)	11 (5.14%)	13 (2.17%)	*0.028*
Neuromuscular disease	29 (3.5%)	11 (5.14%)	18 (3.01%)	0.148
Recent chemotherapy	35 (4.2%)	13 (6.07%)	22 (3.67%)	0.137
Smoker	111 (13.2%)	21 (9.81%)	90 (15.03%)	0.057
Ex-smoker	221 (26.3%)	65 (30.37%)	156 (26.04%)	0.222
Mean number of co-morbidities, median (range)	2 (0–6)	2 (0–6)	1 (0–6)	*0.009*

Values are number (proportion) or median (range). Comparison between survivors and non-survivors was performed using Chi square or Mann–Whitney U test. P-value of less than 0.05 is italic

### AFsepsis calculations suggest low number of deaths directly attributable to sepsis

**Fig. 1:**
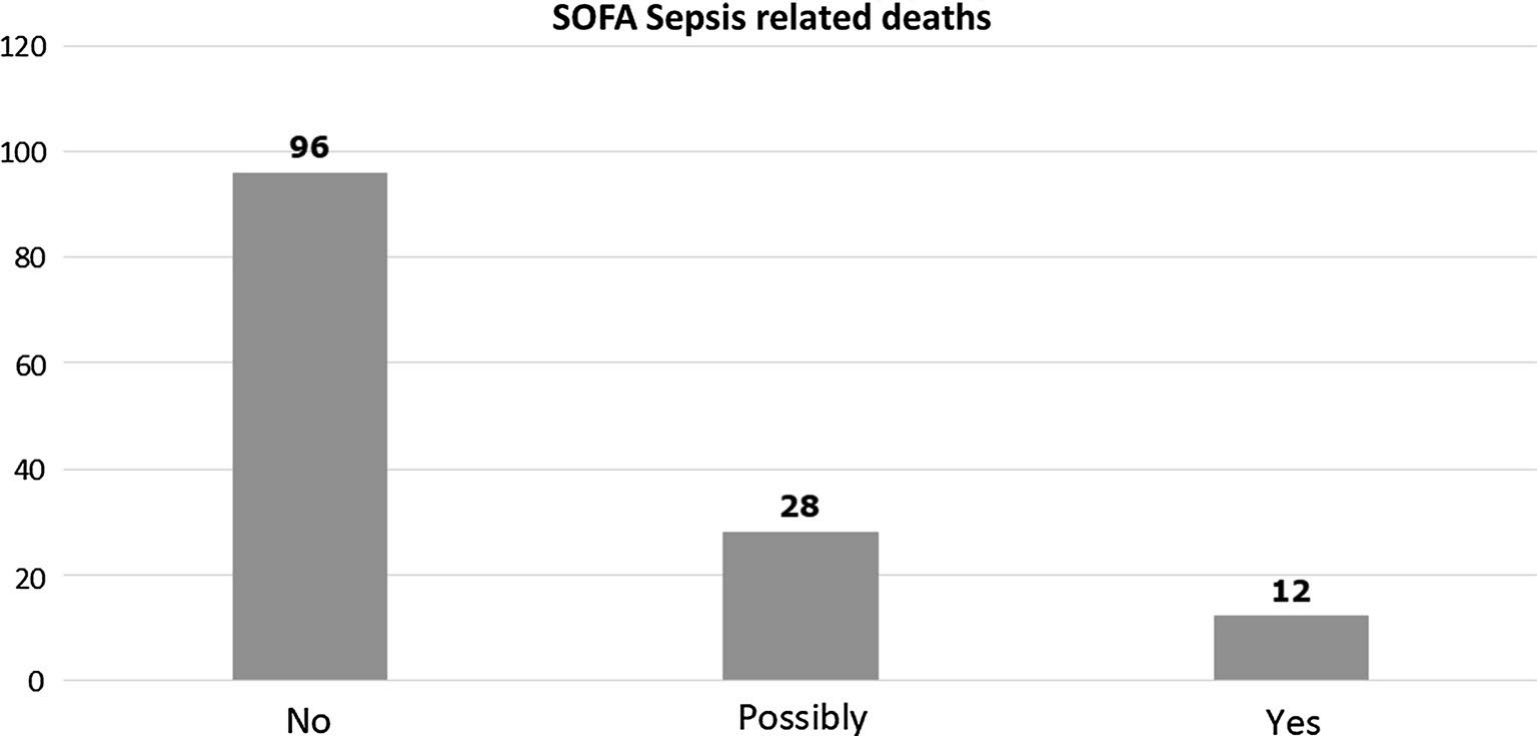
Distribution of patient deaths according to their attribution to sepsis episode

Out of 839 patients, 222 were non-survivors. 166 patients (32.4%) with sepsis (according to SEPSIS-3 criteria) and 56 patients (17.6%) without sepsis died within 90 days. Out of the 166 sepsis deaths 12 (7.2%) could have been directly related to sepsis, 28 (16.9%) possibly related and 96 (57.8%) were not related to sepsis (Fig. 1). Overall AFsepsis was 24.1%.

Upon analysis of the 40 deaths likely to be attributable to sepsis, we found that 31 patients (77.5%) had the Clinical Frailty Score ≥ 6, 28 (70%) had existing DNA-CPR order and 17 had limitations of care orders (42.5%).

### AFsepsis is different for different sepsis screening tools

Out of 394 patients with SIRS ≥ 2, 115 died within 90-day follow-up. Ten deaths (8.7%) could have been directly related to sepsis, 20 (17.4%) possibly related and 72 (62.6%) were not related to sepsis (Additional file 1: Figure S1A). Overall AFsepsis was 26.1%.

Investigating AFsepsis for qSOFA we found that 49 patients with qSOFA ≥ 2 died during the study follow-up. Six deaths (12.2%) could have been directly related to sepsis, 13 (26.5%) possibly related and 21 (42.9%) were not related to sepsis (Additional file 1: Figure S1B). Overall AFsepsis was 38.8%.

### Discussion

To our knowledge this is the first prospective study analysing the attributable fraction of mortality due to sepsis on general wards and ED. We found that the overall AFsepsis in patients fulfilling SEPSIS-3 criteria was 24.1%.

Our analysis shows that burden of sepsis in death may be overestimated. This has important implications for RCTs, as overestimation of AFsepsis could lead to underpowering of the trials and subsequently failure of interventional therapies to show statistical difference in improvement of patient outcomes [3]. Thus, in agreement with previous studies conducted in the intensive care setting we suggest, that AFsepsis should be used in designing such trials rather than the overall mortality in population of patients suffering from sepsis [3].

The most significant variables in patient survival, both in the whole at-risk population and in population of patients who died directly due to sepsis, are patient pre-admission characteristics such as age and patient reserve. This is similar to data by Mahalingam et al. [13] who showed that frailty was associated with increased risk of developing sepsis as well as increased mortality risk after sepsis episode. Strikingly, 28 (70%) of patients who died due to sepsis had DNA-CPR order in place meaning their death was already anticipated by the treating team independently of the current sepsis episode. It is therefore possible that more aggressive treatment could bring more harm than benefit to this group of patients. On the other hand, 12 (30%) of patients whose death was attributable to sepsis did not have the DNA-CPR order in place. We believe that finding a diagnostic tool to identify this subgroup of patients is necessary as early, targeted treatment in the form of the Sepsis Six bundle could have a substantial long-term benefit in their survival [9]. In our study, none of those patients received the complete bundle [7, 8].

Our results indicate, that revisiting the approach to sepsis research is needed [4, 5]. Improvements in sepsis diagnostics are necessary, with more accurate screening tools able not only to identify patients suffering from sepsis but also able to predict patient response to aggressive, sepsis specific management. This would enable clinicians to decide about pursuing aggressive and invasive therapies as opposed to general supportive care. It is also been long recognized that mortality, as an endpoint, presents significant challenges in trial design: its interpretation depends on the time horizon over which mortality is measured; it is a poor tool to use in early phase clinical research to improve selection of the study population; it also provides no insight regarding clinical efficacy in attenuating physiological disturbance [5]. Better diagnostics would improve the understanding of mortality attributable to sepsis. AFsepsis in turn would inform the design of RTCs and provide information used for power calculations and an optimal patient selection. This change of approach to trial design could result in more structured development of therapies for patients suffering from sepsis and also reduce harm from excessive fluid and oxygen administration, antibiotic use and unnecessary testing in patients where either systemic infection is not present, or it is only a bystander of the ongoing disease process [14].

The strengths of our study include participation of centres all across Wales including both academic centres and general district hospitals using prospective data collection methods and providing objective reflection of sepsis prevalence in NHS hospitals. Our study has high internal validity as our previous two studies applied similar methodology [7, 8].

The low proportion of preventable and modifiable elements of sepsis deaths should inform the design of interventional studies. More appropriate identification of patients who could actually benefit from aggressive sepsis specific interventions should be considered not just based on acuity, but also on pre-admission trajectories.

## Limitations

Our study has some limitations. Firstly, we could have missed patients with sepsis who had NEWS below 3 [15, 16]. However, recent data suggest that the NEWS cut-off of 3 may be the most sensitive trigger to screen patients for sepsis outside of the intensive care setting [17]. Score of 3 is also recommended as an escalation trigger by NICE and used in the Sepsis Trust’s Red Flag Sepsis pathways. Secondly, our definition of sepsis-related deaths is arbitrary. Unfortunately, there is no gold-standard, agreed and validated approach to this question and previous studies have noted that relying on death certificates, even when looking at multiple cause of death registries, is likely to produce significant underestimation of sepsis related deaths [18–20]. These studies agree that prospective evaluation of causality would be more appropriate and our prospective studies using multiple sources of information have made this evaluation possible [7, 8, 18–20]. Further validation of our approach is being carried out in separate datasets.

Thirdly, our dataset was a compromise between capturing all possible determinants of sepsis using different screening tools and maintaining simple structure and reliability during data collection.

## Additional file

**Additional file 1: Figure S1.** Distribution of patient deaths according to their attribution to sepsis episode defined by different sepsis definitions.

## Abbreviations

AFsepsis: attributable fraction of mortality due to sepsis
COPD: chronic obstructive pulmonary disease
DNA-CPR: do not attempt cardiopulmonary resuscitation
ED: emergency departments
SIRS: systemic inflammatory response syndrome
SOFA: sequential organ failure assessment
qSOFA: quick sequential organ failure assessment
RCT: randomised controlled trials
