# Supplementary material for: Sepsis-related deaths in the at-risk population on the wards: attributable fraction of mortality in a large point-prevalence study
Source: BMC Res Notes. 2018 Oct 11;11:720. doi: 10.1186/s13104-018-3819-2 (PMC6182791; doi:10.1186/s13104-018-3819-2)
Supplement: Supplementary file 1 — Additional file 1: Figure S1. Distribution of patient deaths according to their attribution to sepsis episode defined by different sepsis definitions. [file 13104_2018_3819_MOESM1_ESM.docx]

Additional file 1: Figure S1. Distribution of patient deaths according to their attribution to sepsis episode defined by different sepsis definitions


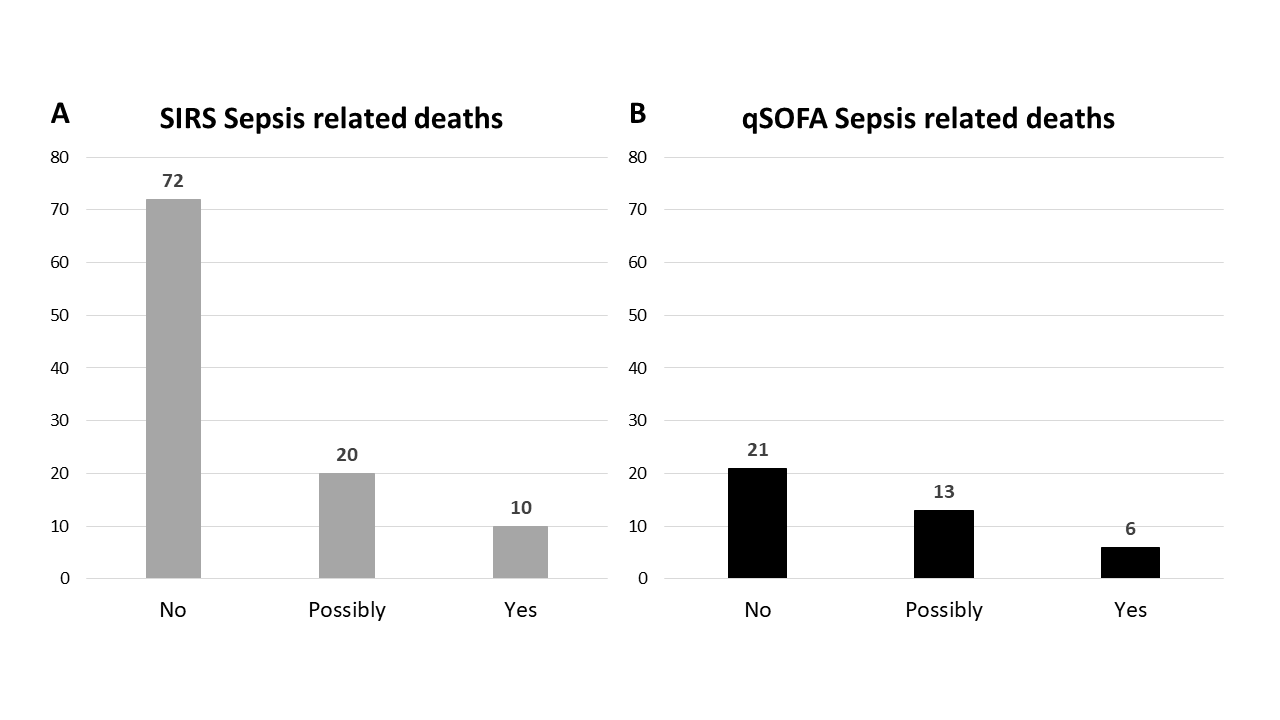


(A) Patients fulfilling SIRS criteria; (B) Patients fulfilling qSOFA criteria.
